# Genetic epidemiology of the Alpine ibex reservoir of persistent and virulent *brucellosis* outbreak

**DOI:** 10.1038/s41598-020-61299-2

**Published:** 2020-03-10

**Authors:** Erwan Quéméré, Sophie Rossi, Elodie Petit, Pascal Marchand, Joël Merlet, Yvette Game, Maxime Galan, Emmanuelle Gilot-Fromont

**Affiliations:** 1 CEFS, INRAE, Université de Toulouse, Castanet-Tolosan, France; 2 ESE, Ecology and Ecosystems Health, Agrocampus Ouest, INRAE, 35042 Rennes, France; 3 Office Français de la Biodiversité, Unité Sanitaire de la Faune, Gap, France; 4 Office Français de la Biodiversité, Unité Ongulés sauvages, Gières, France; 5 Laboratoire Départemental d’Analyses Vétérinaires de Savoie, Chambéry, France; 6 CBGP, INRAE, CIRAD, IRD, Montpellier SupAgro, Univ Montpellier, Montpellier, France; 7 Université de Lyon, VetAgro Sup – Campus vétérinaire de Lyon, Marcy l’Étoile, France; 8 Université de Lyon 1, UMR CNRS 5558 Laboratoire de Biométrie et Biologie Evolutive (LBBE), Villeurbanne, France

**Keywords:** Evolutionary ecology, Immunogenetics

## Abstract

While it is now broadly accepted that inter-individual variation in the outcomes of host–pathogen interactions is at least partially genetically controlled, host immunogenetic characteristics are rarely investigated in wildlife epidemiological studies. Furthermore, most immunogenetic studies in the wild focused solely on the major histocompatibility complex (MHC) diversity despite it accounts for only a fraction of the genetic variation in pathogen resistance. Here, we investigated immunogenetic diversity of the Alpine ibex (*Capra ibex*) population of the Bargy massif, reservoir of a virulent outbreak of brucellosis. We analysed the polymorphism and associations with disease resistance of the MHC Class II *Drb* gene and several non-MHC genes (Toll-like receptor genes, *Slc11A1*) involved in the innate immune response to *Brucella* in domestic ungulates. We found a very low neutral genetic diversity and a unique MHC *Drb* haplotype in this population founded few decades ago from a small number of individuals. By contrast, other immunity-related genes have maintained polymorphism and some showed significant associations with the brucellosis infection status hence suggesting a predominant role of pathogen-mediated selection in their recent evolutionary trajectory. Our results highlight the need to monitor immunogenetic variation in wildlife epidemiological studies and to look beyond the MHC.

## Introduction

The epidemiological dynamics of infectious diseases results from a complex interplay between the pathogen, the host and the environment^1^. Often neglected, inter-individual variation in host susceptibility plays a key role in this dynamics since, depending on their biology (e.g. age, sex), their spatial or social behaviours and/or their genetic characteristics, some individuals contribute far more than others to disease spread^2^. Therefore, identifying individuals and their characteristics that are most responsible for disease transmission is an important step for improving epidemiological surveillance and management of wildlife diseases^3^.

Host genetic and immunogenetic characteristics are rarely investigated in wildlife epidemiological studies despite evidence that inter-individual variation in the outcomes of host–pathogen interactions is at least partially genetically controlled^4^. Pathogen-mediated selective pressures shape the genetic components of host immunity and give rise to inter-individual variation in resistance to infectious diseases^5^. This immunogenetic variation is particularly well documented in humans and domestic animals but much less in wild animals. Furthermore, most studies focused solely on the adaptive branch of the immune system, particularly on the major histocompatibility complex (MHC)^6,7^. Although there is no doubt that MHC plays a key role in individual susceptibility to many diseases^8^, it accounts for only a fraction of the genetic variation in pathogen resistance^5,9^. Before the adaptive immunity intervenes in the host response, other genes involved in the innate immunity such as the Toll-like receptors (TLRs) genes are major players in the host frontline defense against a wide range of microparasites including vectors of zoonotic diseases such *as Coxiella brunetii* (Q fever) or *Borrelia sp*. (Lyme disease)^10–12^.

Genetic variation at the MHC and other immunity-related genes encoding pathogen recognition receptors is shaped by both neutral (i.e. genetic drift, mutation, migration) and selective processes. The relative importance of these evolutionary forces depends on both the population demographic history that influences the strength of the genetic drift, and the (multi)pathogen challenge that determines the type and intensity of selection processes^13^. Populations that went through severe bottlenecks may show low levels of genetic diversity at the MHC (Mainguy *et al*.^14^ on the mountain goat *Oreamnos americanus*; Bollmer *et al*.^15^ on Galapagos penguin *Spheniscus mendiculus*) or TLR loci (Grueber *et al*. 2013 on Stewart Island robin *Petroica australis rakiura*). However pathogen-mediated balancing selection may in some cases buffer the effect of drift to maintain polymorphism^16,17^. Several not-mutually exclusive mechanisms may act in synergy to maintain immunity-related diversity, the three more widespread being: the “negative frequency-dependence”, the “heterozygous advantage” and the “fluctuating selection”. The “negative frequency-dependent selection” (also called rare-allele advantage) hypothesis suggests that the fitness-advantage of alleles is inversely proportional to their frequency in the population as a result of the host-pathogen^18^ or prey-predator co-evolutionary dynamics^19^. The “heterozygote advantage” hypothesis predicts a correlation between individual heterozygosity and fitness or disease susceptibility^20,21^. The “fluctuating selection” hypothesis^22,23^ suggest that population genetic diversity is maintained when spatially and/or temporally varying directional selection occurs^24^.

Here, we investigated the neutral and immunity-related genetic diversity of the Alpine ibex (*Capra ibex*) population from the Bargy area in France. This ibex population from the northern French Alps has undergone a major outbreak of brucellosis (*Brucella melitensis*). Until very recently, European wild ungulates were seen as dead-end hosts for this pathogen^25^ and all previous outbreaks of brucellosis in wild ruminants spontaneously faded out^26–28^. However, the situation in Bargy is alarming since the disease has affected an unprecedented high proportion of the population (38% in 2013, 95% CI [28.2; 47.8]; n = 77). Brucellosis persisted at high levels in the following years despite intense culling of animals which led to the removal of 40% of the population (see Marchand *et al*.^29^ for details). Beyond the conservation and ethical issues, this outbreak has raised public health and economic concerns since, for the first time, cattle and humans were infected by the wildlife reservoir^30^. In this context, it was critical to identify the drivers of pathogen persistence in this population and individual characteristics favoring its spread. Previous works showed that host-infectiousness vary significantly across age and sex and among socio-spatial units within the Bargy massif^29^. However, genetic factors of susceptibility to brucellosis in Alpine ibex have never been investigated while several associations with innate gene polymorphism have been discovered in domestic ungulates^31,32^.

The genetic issue is particularly pertinent in Alpine ibex since this species underwent a major bottleneck during the XIX^th^ century due to overhunting, followed by several successive bottlenecks during its reintroduction history across the Alpine arc. Most current populations have been re-established few decades ago from a limited number of founder individuals^33^. This reintroduction history has had drastic genetic consequences: all Alpine ibex populations exhibit a particularly low level of neutral and MHC genetic diversity and a high level of inbreeding^33,34^ with a direct influence on traits related to survival and reproductive success such as body mass or parasite load^35^.

The first aim of this study was to assess the neutral and immunity-related genetic variation of the Bargy population. We screened the neutral microsatellite diversity to test the hypothesis that the intrinsic susceptibility to brucellosis of ibex at Bargy results from a particular low genetic diversity in comparison with other reintroduced ibex populations. We also evaluated the polymorphism of the commonly used *Mhc-Drb* as well as four immunity-related genes (*Tlr1*, *Trl2*, *Tlr4*, Solute Carrier family 1 hereafter *SLC11A1*) involved in the innate immune response to *Brucella sp*. in domestic ungulates^31,32,36^. The second objective was to investigate associations between individual brucellosis infection status and genetic/immunogenetic profile. We first assessed the role of multi-locus heterozygosity at neutral microsatellites as a proxy of inbreeding (heterozygosity-fitness correlation). We then looked at potential associations with the heterozygosity status (heterozygous advantage hypothesis) and/or specific haplotype of immunity-related genes. In both domestic and wild ungulates, brucellosis is well-known to cause abortion and reproductive failures^37,38^ with known impacts on individual fitness. Therefore, these associations may reflect pathogen-mediated balancing or directional selective pressure on immunity-related gene polymorphism.

## Results

### Neutral genetic diversity

The tests for Hardy-Weinberg equilibrium, after correction for multiple testing, revealed that SR-CRSP07 STR locus showed a significant excess of homozygotes. This marker was thus removed from further analysis. H_E_ was 0.43 ± 0.17 (mean ± standard error) and mean N_A_ was 3.12 over all microsatellite loci. Neutral variation did not vary significantly before (H_E_ = 0.42 ± 0.16, sN_A_ = 2.64 ± 0.79) and after (H_E_ = 0.42 ± 0.17, N_A_ = 2.78 ± 0.84) the culling operations that occurred in 2013–2015 (Student’s t-test, df=109, p = 0.93 for H_E_; p = 0.39 for sNA).

### Immunity-related gene polymorphism

The Illumina sequencing of the exon 2 of the *Mhc-Drb* gene on 144 individuals revealed a unique haplotype in Bargy corresponding to CaIb-DRB*1 haplotype, previously isolated by Schalschl *et al*.^39^ and Grossen *et al*.^40^. The Sanger sequencing of the three *Tlr* genes generated an average of 2,169 bp of sequence covering almost the entire coding region of the genes (92% on average). *Tlr* genes exhibited seven SNPS among which five were non-synonymous. We isolated 1 SNPs for *Tlr1* and revealed two functional haplotypes (i.e. encoding different amino acid sequences) including a rare haplotype (5%) observed in only 14 individuals. The *Tlr2* gene exhibited three SNPs (two non-synonymous) and showed three functional haplotypes with a common haplotype (*Tlr2a* = 72%) and two haplotypes with low frequencies (*Tlr2b* = 15% and *Tlr2c* = 13%). Lastly, we found three SNPs (two non-synonymous) in *Tlr4* gene and identified four functional haplotypes among which three haplotypes showed similar moderate frequencies ranging between 25% and 35% while the fourth one was rare (9%). Expected heterozygosity (H_E_) (given differences in haplotype frequencies) varied greatly among *Tlrs* ranging from 0.09 for *Tlr1* to 0.71 for *Tlr4* (Table 1). The *SLC11A1* gene showed two alleles (“A324” and “A330” hereafter) with frequencies of 75% and 25% respectively (H_E_ = 0.37).

**Table 1:** Patterns of genetic diversity for immunity-related genes and neutral loci.

Gene	*2n*	*N*_*H*_	*H*_*E*_	*F*_*IS*_
*SLC11A1*	262	2	0.37	−0.07
*Tlr1*	146	2	0.09	−0.05
*Tlr2*	142	3	0.45	−0.03
*Tlr4*	138	4	0.71	0.06
Neutral microsatellites	237	3.12	0.43	0.01

*2n* is the sample size, *N*_*H*_ is the number of haplotypes/alleles, *H*_*E*_ is the expected heterozygosity and *F*_*IS*_ is the departure from Hardy-Weinberg equilibrium within populations.

### Genetic effects on brucellosis infection

**Figure 1:**
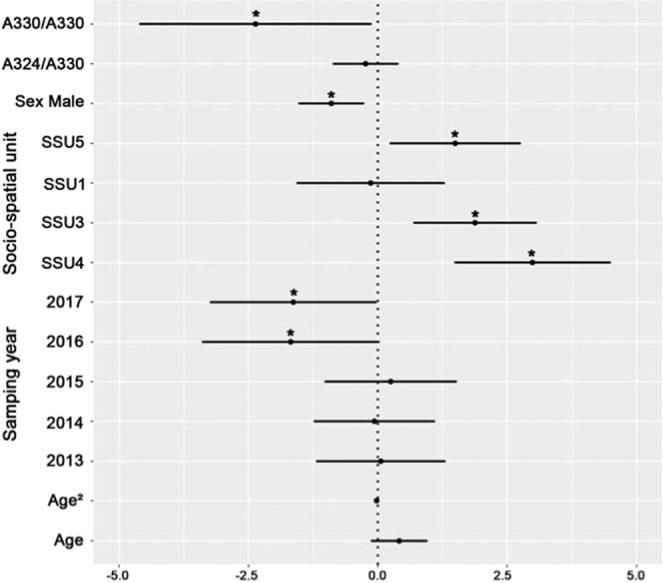
Genetic association between *Slc11A1* and brucellosis serological status. Model averaged parameter estimates and their 95% confidence intervals for the *Slc11A1* genotype, biological (Age, Sex) and environmental factors (Year, Socio-spatial units). The symbol * indicates a parameter with a significant effect. Females from SSU2 sampled in 2012 were used as the reference category.

**Figure 2:**
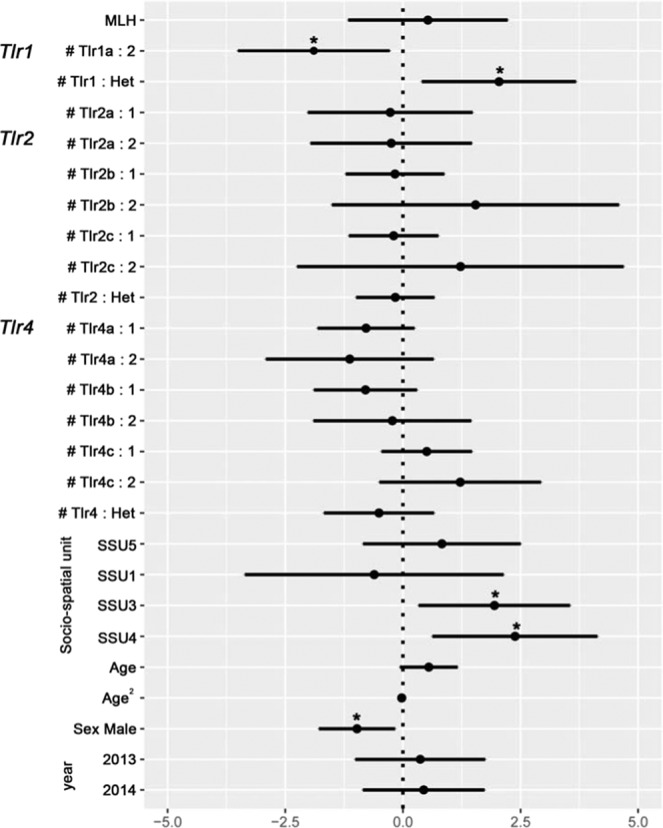
Genetic effects of neutral multi-locus heterozygosity (*MLH*) and *Tlr* polymorphism on brucellosis serological status. Model averaged parameter estimates and their 95% confidence intervals for the *MLH* (Multi-locus heterozygosity estimated from neutral microsatellite loci), *Tlr* (*Tlr1, Tlr2, Tlr4*) number of copies (0, 1 or 2) of each haplotype with frequency >10%, *Tlr* heterozygosity status (*Het*), biological (Age, Sex), and environmental factors (Year, Socio-spatial units). The symbol * indicates a parameter with a significant effect. Females from SSU2 sampled in 2012 were used as the reference category.

Out of the 237 ibex from the Bargy massif that were first tested, 89 were found seropositive to brucellosis (37%). The probability to be seropositive was lower in males than in females and reached a maximum in the core area of the massif (SSU3, SSU4) (see Tables S4,S5, Figs. 1,2). We observed a slight temporal decrease of the seroprevalence over the monitoring period (only significant for the dataset covering the whole 2012–2017 study period). Brucellosis seropositivity also tended to increase with age but 95% confidence intervals of the effect size did not exclude zero (Average Effect Size = 0.41 [95% CI: −0.13,0.95]) (Fig. 1, Table S4). We did not find any significant effect of the multi-locus heterozygosity (MLH) on the individual serological status (general effect) (Fig. 2, Table S5) but we observed associations with immunity-related genes. First, we found a strong relationship between *SLC11A1* genotype and brucellosis status (Fig. 1). Prevalence decreased with the number of copies of the less frequent allele “A330”. This effect was particularly marked for individuals carrying two copies of this allele (homozygous individuals) (AES = −2.36 [−4.60, −0.13]) but less clear for heterozygous individuals (AES = −0.24 [−0.86, 0.39]), which suggests that “A330” is recessive. We also revealed a significant association with *Tlr1* genotype (Fig. 2, Table S5): homozygous individuals carrying two copies of the frequent haplotype (*Tlr1a*) had a lower probability to be seropositive than heterozygous individuals (AES = −1.90 [−3.52, −0.29]). *Tlr1* heterozygous status was also associated with ibex brucellosis status (Table S5) but this most likely result from the systematic presence of the *Tlr1b* haplotype in *Tlr1* heterozygous ibex. We did not find any effect of *Tlr2* and *Tlr4* haplotypes (directional selection) or of the heterozygous/homozygous status (heterozygote advantage) (Table S5).

## Discussion

The aim of this study was to assess the neutral and immunity-related genetic diversity of the Alpine ibex population of the Bargy massif, wildlife reservoir of a persistent and virulent outbreak of brucellosis. As expected, this population established a few decades ago from a very limited number of founders (<15 ibex) shows very low genetic variation at neutral microsatellite loci and exhibits a single MHC Class II haplotype similarly to most ibex populations of the Western Alps^40^. By contrast, other immunity-related genes involved in the innate immunity such as *Tlr* genes (*Tlr1*, *Tlr2* and *Tlr4*) and *Slc11A1* have maintained polymorphism despite serial founder events occurring throughout the Alpine ibex reintroduction history. We revealed significant associations between the polymorphism of some of these genes (*Tlr1* and *Slc11A1*) and *Brucella* infection status, which may suggest a role of pathogen-mediated selection in their recent evolution.

### Low neutral genetic diversity and absence of genome-wide effect on brucellosis infection

With an expected heterozygosity (H_E_) of ~40% and an average number of alleles (MNA) per microsatellite loci <4, the ibex population of Bargy shows particularly low levels of microsatellite genetic variation, compared to values reported in goat breeds (MNA = 5–9 alleles, H_E_ = 0.60–0.76, Canon *et al*. 2006) and in other wild European ungulate populations (e.g. H_E_ = 0.56–0.67 in roe deer *Capreolus capreolus*, Quéméré *et al*.^17^; H_E_ = 0.58–0.67 in red deer *Cervus elaphus*, Zachos *et al*.^41^). However, these differences may arise because the microsatellite loci used in the different species underwent different ascertainment bias. Our results are consistent with the levels previously observed in other Alpine ibex populations reintroduced in Switzerland during the XX^th^ century^42^ and in particular, for the Mont Pleureur population (H_E_ = 0.42) from which the Bargy founders came from. Low genetic diversity might reduce individual fitness and population demographic performance due to reduced evolutionary potential^43,44^. For example, in the Gran Paradiso ibex population, there is a direct relationship between multi-locus microsatellite heterozygosity, male body mass and gastrointestinal nematode infestation (fecal egg counts) (i.e. Heterozygosity-fitness correlation)^35^. In our case, we did not observe any relationship between the genome-wide heterozygosity (MLH) of ibex and their *Brucella* infection status. This is in agreement with the recent study of Brambilla *et al*.^45^ that did not find any effect of neutral heterozygosity on infectious keratoconjunctivitis symptoms, another bacterial disease recurrently affecting Alpine ibex populations. Similar low levels of neutral genetic diversity were observed in other brucellosis-infected ibex populations (Grand Paradiso) in which the prevalence level has remained low and infection vanished spontaneously^26^. Therefore, contrary to our hypothesis, the low level of neutral diversity at Bargy is in itself not sufficient to explain its potential higher sensitivity to brucellosis compared to other ibex populations. However, a growing number of studies illustrated the poor predictive power of microsatellite loci to evaluate functional diversity and showed the importance of accounting for immunogenetic variation to get a better understanding of the population’s adaptive response potential to pathogen-mediated selection^46,47^.

### Maintenance of immunity-related gene polymorphism in Alpine ibex

Our study is the first to our knowledge to reveal immunity-related polymorphism in non-MHC genes in Alpine ibex. Previous work on ibex focused exclusively on MHC Class II genes^45,48^ that accounts for only a fraction of the genetic variation in pathogen resistance^5,9^. MHC class II molecules principally bind exogenous antigens and are primarily involved in the immune response to extracellular pathogens^49^. By contrast, genes encoding toll-like receptors (*Tlrs*) play a fundamental role in vertebrate innate immune defense against intracellular and extracellular micropathogens including viruses, bacteria, protozoa or fungi^50^. In particular, these genes are involved in the recognition and immune response to pathogenic bacteria that recurrently cause outbreaks such as *Mycoplasma* sp. (causing pneumonia or infectious kerotoconjunctivitis infections)^51,52^ or *Brucella abortus*^53,54^. Contrary to our expectation and despite serial founder events throughout the species reintroduction history, the three studied *Tlr* genes and the *SlC11A1* have all maintained several haplotypes while the MHC Class II DRB (second exon) is monomorphic in Bargy. There are very few studies that investigate *Tlr* polymorphism in ungulates populations: levels of *Tlr* haplotype diversity in Bargy ibex (N_H_ = 3–5, H_D_) appears similar to those reported for the same genes in roe deer populations (N_H_ = 3–5)^17^, domestic goat (N_H_ = 1–4)^55^ or cattle (N_H_ = 2–5)^56^. *Tlr4* showed four functional haplotypes (i.e. encoding different amino-acid sequences) including three alleles with moderate to high frequencies (>25%). This balanced polymorphism which contrasts with the very low neutral and MHC diversity in Bargy, suggests that balancing pathogen-mediated selection may have favoured the maintenance of genetic variation as showed in other free-ranging animal species^12,57,58^. Furthermore, it is worth noting that the presence of balanced polymorphism does not mean that the gene is involved in brucellosis resistance, which can be demonstrated only by studying associations between immunogenetic patterns and pathogen prevalence^59^.

### Genetic factors of resistance to Brucella melitensis infection in Alpine ibex

In contrast to Brambilla *et al*.^35^, we did not find evidence of correlation between multi-locus heterozygosity at neutral microsatellites and brucellosis infection status (“general effect” hypothesis). This means that a higher individual multi-locus heterozygosity (a proxy of the level of inbreeding) does not confer a resistance advantage regarding *Brucella* infection. In contrast, we found associations between single immunity-related genes (here *Tlr1*, *Slc11A1*) and ibex infection status hence supporting the “local effect” hypothesis in agreement with Brambilla *et al*.^45^. These authors observed a lower susceptibility of MHC *Drb* heterozygous to infectious keratoconjunctivitis (heterozygote advantage hypothesis) while we revealed here an effect of specific alleles. Ibex carrying two copies of the less frequent allele of the *Slc11A1* gene were less likely to develop the *Brucella* infection, which suggests that this “resistance allele” is recessive. Associations between a specific *Slc11A1* allele and *Brucella* infection has been already reported in domestic goat^36^ and water buffalo^31^. Moreover, in an *in-vitro* experiment on water buffalo’s monocytes infected by several *Brucella* species including *B. melitensis*, Borriello *et al*.^60^ demonstrated that some *Slc11A1* genetic variants confer a higher *Slc11A1* mRNA expression and a higher ability in controlling the intracellular replication of the *Brucella*. The “resistant allele” (A330) showed similar frequency in the Bargy and Aravis populations (25% and 21% respectively) but is much more frequent in other Alpine populations (e.g. 54% in Belledonne massif (northern French Alps), 40% in Grand Paradiso National Park – results not shown). Further comparison with other recently reintroduced ibex populations should be performed to investigate whether the particularly low frequency of this resistant variant in Bargy may have favored the emergence of this brucellosis outbreak together with other, non-genetic factors such as spatial ibex distribution and abundance.

Our results also suggest that the most frequent haplotype of *Tlr1* (96%) conferred a resistance advantage against *Brucella* infection. However, this resistance advantage is relative: although 92% of ibex in Bargy are homozygous for this allele, 37% are still seropositive. Therefore, individuals that carry out this allele have a lower probability to get infected after a contact with the bacteria, but do not show a complete resistance. Such relationship between *Tlr1* genotype and *Brucella* infection has been already evidence in Cattle by Prakash *et al*.^32^. *Tlr1* interact with *Brucella* sp. by recognizing specific pathogen-associated molecular patterns. Individuals carrying the rare allele (*Tlr1b*) may have a decreased ability to recognize *Brucella* and thus to trigger an adequate immune response. Further work is needed to clarify the functional role of these immunity-related genes against brucellosis in Alpine ibex and in particular how these polymorphisms affects the detection and the replication of the bacteria (see Borriello *et al*.^60^). Particularly, *in vitro* studies of ibex white blood cells and their response to *Brucella*^36^ would help to confirm a variation linked to immune genes and to identity cellular pathways to resistance.

In agreement with our hypothesis and the literature on domestic ungulates, we showed significant associations between immunity-related genes (specific alleles *Slc11A1* and *Tlr1*) and brucellosis infection status in Alpine ibex. However, this does not in itself provide evidence for directional pathogen-mediated selection because we did not actually demonstrate that these alleles confer a selective advantage. Seropositive ibex were euthanized, so there is no data available on the impact of *B. melitensis* infection on survival and reproduction rates in this population. However, brucellosis is well-known to cause abortion and reproductive failures in other domestic and wild ungulates^37,38^. So we can reasonably hypothesize that *B. melitensis* infection actually affects the fitness of ibex in Bargy and may ultimately lead to selection on these genes.

### Management implications

Genetic monitoring is widely recognized as a valuable tool for the management and conservation of populations^61^. In particular, access to population genetic parameters may help to better understand the ecology of host/pathogen interaction and thus better manage wildlife diseases^62,63^. Most genetic studies used microsatellites and MHC Class II markers to investigate population adaptive diversity and tolerance or resistance to diseases^8^ with very few exceptions^64^. However, Alpine ibex exhibit a very low neutral genetic diversity and a unique MHC Class II DRB exon 2 haplotype in most recently introduced populations. Furthermore, most potentially zoonotic diseases affecting wild ungulate populations and notably Alpine ibex (e.g. Q fever, tuberculosis, chlamydiosis) involve micropathogens (virus, bacteria, protozoa) and MHC Class II often plays a minor role in the immune response against these pathogens^49^. Our study highlights the need to look beyond MHC class II and to explore the diversity of other candidate immunity-related genes^5^, in particular MHC class I, cytokine and Toll-like receptors genes that are centrally involved in the innate immune response against micro pathogens in humans and domestic species.

In a conservation framework, our results call for immediate management actions to increase the neutral and immunity-related diversity of the Bargy ibex population, whose census size had been halved (~300 individuals in 2014–2017 against ~600 in 2013) over the monitoring period. Translocations of unrelated individuals from the Gran Paradiso source population or from other genetic units of Alpine ibex^34,65^ is recommended to prevent inbreeding depression and increase both genome-wide^35,45^ and immunity-related diversity^47^. Further, these results raise the question of the interplay between sanitary management and population genetic structure. Here, the test-and-cull management procedure was expected to affect the genetic structure of the population, by selectively eliminating infected individuals carrying specific alleles. In the same line, some authors suggested that artificial selection of specific *Slc11A1* or *Tlr* genotypes in breeding programs may help to increase population natural resistance and limit spread of brucellosis in domestic ungulates^31,32^. However, it is important to keep in mind that natural populations are exposed to multi-pathogen pressures (e.g. brucellosis and Q fever in the Bargy population) and that most immunity-related genes are involved in the immune response against several pathogens. Therefore, artificially selecting a specific genotype to increase population resistance to a pathogen may lead to the loss of key haplotypes in the host-control of other diseases, particularly in species with very low genetic diversity. Incorporating data on immunogenetic variation in epidemiological models should help to predict how variation in allele frequency may affect the spread dynamics of the disease, and how disease management may affect genetic structure of the population.

## Methods

### Study site, population and sampling

**Figure 3:**
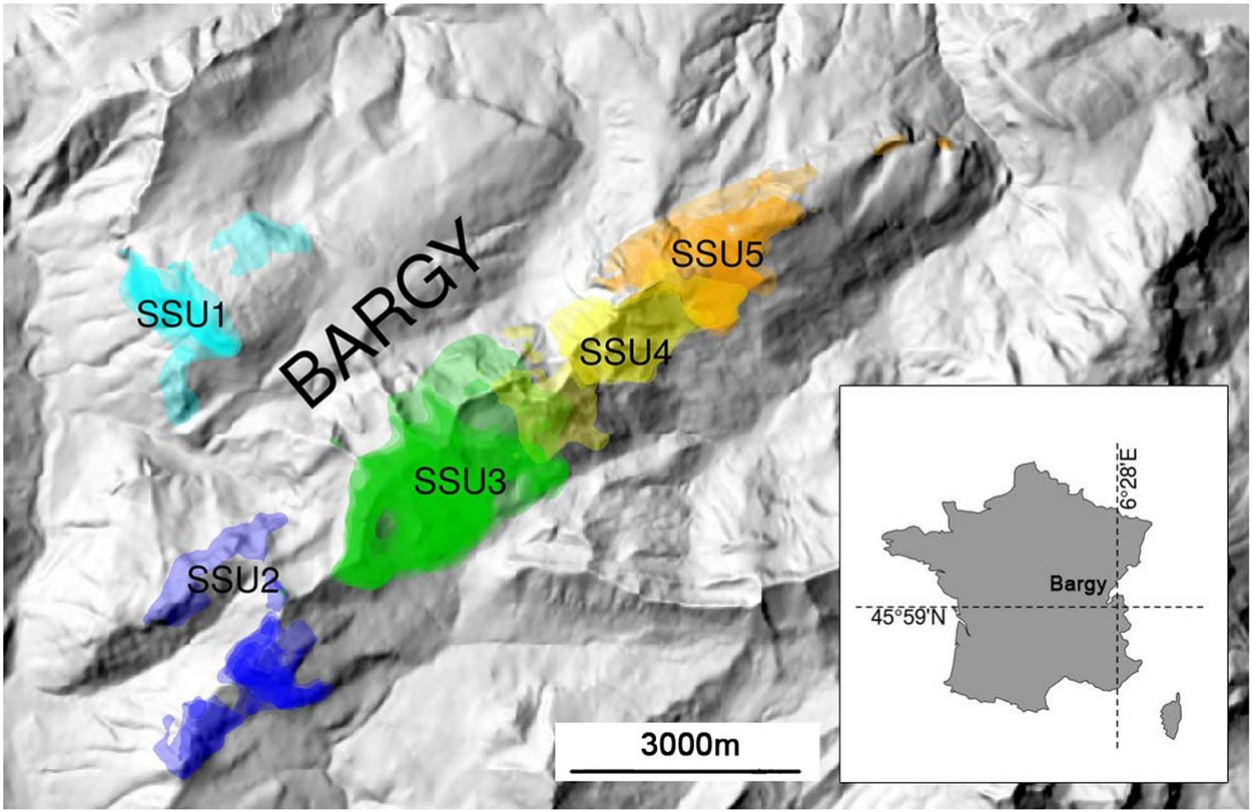
Location of the five socio-spatial units (SSU) of female Alpine ibex. These five socio-spatial units correspond to the best number of spatially-segregated groups as determined by hierarchical classification methods on distances between individuals measured as overlap between annual home ranges of GPS-collared females. Adapted from Marchand *et al*.^29^ (CC by 4.0 International license).

Alpine ibex were reintroduced in the Bargy Massif (France) during three release events in 1974 and 1976 comprising a total of 14 released individuals (i.e. 6 males and 8 females) translocated from the Mont Pleureur population in Switzerland^66^. Since the discovery of the outbreak in 2012, the population has been continuously monitored by Capture-Mark-Recapture (CMR)^29^. Using GPS data on marked individuals, Marchand *et al*.^29^ demonstrated that females are structured in five distinct socio-spatial units (SSU1-SSU5, Fig. 3), whereas males are prone to move between units especially during the mating period^29^. Here, we studied the genetic and immunogenetic characteristics of 262 ibex captured between 2012 and 2017 by the French Hunting and Wildlife Agency in accordance with legal and ethical regulations (French environmental code, 2005, 2006; Préfecture de Paris, 2009; Préfecture de la Haute-Savoie, 2013, 2015a,b; French Minister of Ecology Sustainable Development and Energy, 2014). Animals were captured by dart-gun xylazine-ketamine anesthesia (Rompun®, Bayer, Leverkusen, Germany and Imalgène®, Merial, Lyon, France; 100 mg/individual) following protocols described in Lambert *et al*.^67^. During captures, test-and-cull was implemented on the basis of serological tests (see below for further details on the serological method) as part of management measures decided by the French Authorities (Hars *et al*., 2013). Seronegative individuals were marked and released while seropositive ones were euthanized. All captured animals were handled by professionals from the French Game and Wildlife management agency (Office National de la Chasse et de la Faune Sauvage, Office Français de la Biodiversité) according to the ethical conditions detailed in the specific accreditations delivered by the Préfecture de Paris (prefectural decree 2009–014), by the French Minister of Ecology, Sustainable Development and Energy (Ministerial Orders of February 11, 2014) and by the Préfecture de la Haute-Savoie (prefectural decree 2015062- 0018) in accordance with the French environmental code (Art. R421-15 to 421-31 and R422-92 to 422-94-1). Euthanasia was performed by veterinarians in accordance with the requirements of these accrediting authorities, and slaughtering operations were performed in accordance with accreditations delivered by the Préfecture de la Haute-Savoie (prefectural decrees 2013274-0001, DDT-2015-0513, DDT-2016-0933, DDT-2017-1003). We hence confirm that we treated animals in accordance with relevant guidelines and regulations and that our protocols were approved by the National Committee for Nature Protection (CNPN in French), a group of independent experts elicited by the ministry of the environment and in charge of evaluating capture/destruction of the protected species.

In addition to CMR monitoring, French Authorities implemented selective culling of animals with observed clinical signs and mass indiscriminate slaughter of individuals of five years and more: culling operations occurred between autumn 2013 and spring 2014 (N = 256 killed) and then in autumn 2015 (N = 70). The population size dropped from 567 individuals (95% CI: [487–660]) in 2013 to 310 [275–352] in 2014 and 277 [220–351] in 2015^29^.

### Pathogen screening

Brucellosis serologic status of individuals was evaluated using a range of serological tests^68^. In particular, since 2014, the rapid Laminar Flow Immune-chromatographic Assay (LFIA) (Rapid G.S. Brucella Abtest, Bionote, Gyeonggi-do, Rep. of Korea) was performed in the field. This test had been validated on ibex samples by the EU/OIE/FAO and National reference laboratory (ANSES, 2014). Further details on the validation method and the congruence of the FLIA with reference tests (Rose Bengal Test, Complement Fixation Test, iELISA) were provided in Anses (2014). Seropositive ibex were considered as infected by *B. melitensis*, but not necessarily infectious at the time of capture^67^.

### Microsatellite and immunity-related loci genotyping

DNA was extracted from blood using the DNeasy Blood and Tissue kit (QIAGEN). All individuals were genotyped at 25 polymorphic putatively neutral microsatellites^42^ (see list and primers in Table S1). We carried out genotyping replicates for about 25% of the samples to ensure the reliability of the genotyping. Micro-Checker 2.2.3^69^ was employed to assess the frequency of null alleles and scoring errors (stuttering or large allelic dropout). The same individuals were also typed at a microsatellite located at the 3′UTR of the Solute Carrier Family 11 member A1 (*SLC11A1*) gene using the following primers: Fw- TCTGGACCTGTCTCATCACC and Rv- ACTCCCTCTCCATCTTGCTG^70^. The *SLC11A1* gene, formerly known as natural resistance-associated macrophage protein 1 (NRAMP1) gene, is involved in the innate resistance to intra-cellular pathogens^71^. Polymorphism in microsatellites of the 3′ UTR of the gene were associated with resistance to *Brucella* infection in water buffalo *Bubalus bubalis*^36^ and in the domestic goat^31^, a species closely related to the Alpine ibex. In particular, some *SLC11A1* genotypes have a higher ability of controlling the intracellular replication of *Brucella in vitro*^60^.

We also genotyped *Mhc-Drb* and *Tlr* genes for a subset of the individuals (N = 146) sampled between 2012 and 2014. The second exon of the *Mhc-Drb* class II gene encoding the ligand-binding domain of the protein was amplified and sequenced using Illumina MiSeq system following the procedure detailed in Quéméré *et al*.^17^. Haplotypes and individual genotypes were identified using the SESAME barcode software^72^. *Tlr* genes (*Tlr1*, *Tlr2* and *Tlr4*) were genotyped using the two-step procedure described in Quéméré *et al*.^17^. A pilot study on 30 individuals was first performed to identify polymorphic sites (SNPs). We screened almost the entire coding region of the three *Tlr* genes (92% in average) including the leucine-rich extracellular region of receptors involved in antigen-binding. Details on primer sequences and accession numbers are provided in Table S2. SNPs were then genotyped for 146 individuals using the KASPar allele-specific genotyping system provided by KBiosciences (Hoddesdon, UK). Details on SNP position and codon change can be found in Table S3. Haplotypes were then reconstructed from the phased SNPs using the procedure implemented in DNASP v5^73^.

### Genetic diversity

The genetic diversity of both microsatellites and immunity-related genes was evaluated by calculating the expected heterozygosity (H_E_) and the number of alleles/haplotypes (N_A_) as implemented in GENETIX software v4.05.2^74^. For each locus, a test for Hardy–Weinberg proportions was performed based on 1000 permutations. To allow comparison with other ibex populations^42^ and between the two monitoring periods (before and after culling operations), we used FSTAT version 2.9.3^75^ to calculate the allelic richness (sNA), a standardized measure of number of alleles corrected for differences in samples size. Lastly, standardized multi-locus heterozygosity at neutral microsatellites (MLH) was calculated for each individual as the ratio of its heterozygosity to the mean heterozygosity of the population^76^. Our set of microsatellite loci showed significant identity disequilibrium (g2 ± SD = 0.011 ± 0.0065, p = 0.007), (e.g. a measure of covariance in heterozygosity), a condition for detecting heterozygosity-fitness correlations. Both MLH and g_2_ were calculated using the R package inbreedR^77^. To assess whether management operations (i.e. removal of 40% of the population between 2013 and 2015) had led to a decrease of the genetic diversity, we performed a comparison (using Student’s t-tests) of the allelic richness (sN_A_) and expected heterozygosity (H_E_) of ibex sampled before (between autumn 2012 and autumn 2013) and after (years 2016–2017) the slaughtering operations that occurred between autumn 2013 and autumn 2015.

### Genetic association with brucellosis infection

We analyzed the relationships between the serological status of ibex and neutral or immunity-related gene variation using generalized linear models (GLM) of the binomial family (using a logit link function). The serological status of each individual was coded as 1 (positive, considered to have been exposed and not resistant) or 0 (negative, considered to have been either non-exposed or exposed but resistant).

We fitted models testing for the effect of genetic effects, while taking into account other biological and environmental factors that possibly affect pathogen exposure. We tested the “heterozygosity fitness-correlation” hypothesis (i.e. “general effect”) by fitting individual standardized multi-locus microsatellite heterozygosity (MLH) as a fixed effect. For each immunity-related gene, we tested the “heterozygote advantage hypothesis” by fitting a binary fixed effect (heterozygote *vs* homozygote) and the “haplotype advantage” hypothesis by considering associations between the infection status and the number of copies (0, 1 or 2) of *Tlr* or *Slc11A1* haplotypes with frequency >10%. Concerning non-genetic factors, following Marchand *et al*.^29^, we included the effects of ibex sex as a categorical variable and age as a continuous variable in years (linear and quadratic terms). We also fitted a “year” effect as a categorical variable to test for varying epidemiological situations across years and a “location” effect because seroprevalence has been shown to vary among socio-spatial units^29^.

We used a multimodel inference approaches to establish which explanatory variables had an effect, averaged over plausible models^78–80^. The most complete models used in the inference included the effects of genetic effects hypotheses, age sex, year and location. We compared the maximal model with all its sub-models (See Tables S6 and S7 for the full lists of model tested). The effect of *Slc11A1* and *Tlr* genes were evaluated in separate analyses because of different samples sizes (262 and 146 individuals respectively). We conducted an initial exploration of our data to ascertain their distribution and spread, to identify outliers and examine relationships between variables^81^. We used variance inflation factors (VIF) to assess which explanatory variables were collinear, and should be retained in the analyses (i.e. VIF values <3)^81^. To minimize multicollinearity, the effects of the most frequent haplotype of a gene and its heterozygosity status were evaluated in separate models.

Model averaged parameter estimates are provided with their unconditional standard errors (SE) and 95% confidence intervals, after averaging model with ΔAIC < 7 (relative to the best model) as recommended by Burnham *et al*.^79^ and using the “zero method” (i.e. a zero is substituted into models where the parameter is absent). Analyses were performed using the package lme4^82^, MUMIN v1.7.7^83^ and AICcmodavg v1.25^84^ in R version 3.3.3 (R Development Core Team, 2017).

## Supplementary information

Table S1— S5

Table S6.

Table S7.

## Data Availability

Haplotype DNA sequences: primers and Genbank accessions are in Tables S1,S2. SNP positions and characteristics are in Table S3. Genotypes of all individuals at each microsatellite and immunity-related locus are available from the corresponding author upon request to anyone who wishes to repeat our analyses or collaborate with us.
