# Supplementary material for: Genetic epidemiology of the Alpine ibex reservoir of persistent and virulent brucellosis outbreak
Source: Sci Rep. 2020 Mar 10;10:4400. doi: 10.1038/s41598-020-61299-2 (PMC7064506; doi:10.1038/s41598-020-61299-2)
Supplement: Supplementary file 1 — Table S1— S5 [file 41598_2020_61299_MOESM1_ESM.docx]

**Genetic epidemiology of the Alpine ibex reservoir
of persistent and virulent *brucellosis* outbreak**

Erwan Quéméré^1^, Sophie Rossi^2^, Elodie Petit^3^, Pascal Marchand^3^, Joël Merlet^1^, Yvette Game^4^, Maxime Galan^5^, Emmanuelle Gilot-Fromont^6, 7^

^1^CEFS, INRA, Université de Toulouse, Castanet-Tolosan Cedex F-31326, France

^2^ONCFS, Unité Sanitaire de la Faune, Gap

^3^ONCFS, Unité Faune de Montagne, Gières

^4^Laboratoire Départemental d’Analyses Vétérinaires de Savoie, Chambéry
^5^Univ. Montpellier, F-34988, Montferrier-sur-Lez Cedex, France

^6^Université de Lyon, VetAgro Sup – Campus vétérinaire de Lyon, Marcy l’Étoile

^7^Université de Lyon1, UMR CNRS 5558 Laboratoire de Biométrie et Biologie Evolutive (LBBE), Villeurbanne

Corresponding authors: Erwan Quéméré, erwan.quemere@inra.fr.
Phone: (+33) 561 285 497. Fax: (+33) 561 285 500.

Supplementary material

Table S1. List of primers and primers sequences.

| Marker name | Gk Acc NB | Primer A (5'-3') | Primer B (5'-3') |
| --- | --- | --- | --- |
| BM1225 | G18419 | TTTCTCAACAGAGGTGTCCAC | ACCCCTATCACCATGCTCTG |
| BM1258 | G18385 | GTATGTATTTTTCCCACCCTGC | GAGTCAGACATGACTGAGCCTG |
| BM1818 | G18391 | AGCTGGGAATATAACCAAAGG | AGTGCTTTCAAGGTCCATGC |
| BM302 | L12559 | GAATTCCCATCACTCTCTCAGC | GTTCTCCATTGAACCAACTTCA |
| BM415 | G18413 | GCTACAGCCCTTCTGGTTTG | GAGCTAATCACCAACAGCAAG |
| BM4208 | G18509 | TCAGTACACTGGCCACCATG | CACTGCATGCTTTTCCAAAC |
| BM4505 | G18511 | ATCTTCACTTGGGATGCAGG | TTATCTTGGCTTCTGGGTGC |
| CSSM47 | U03821 | CTGGGCACCTGAAACTATCATCAT | TCTCTGTCTCTATCACTATATGGC |
| ETH10 | Z22739 | GTTCAGGACTGGCCCTGCTAACA | CCTCCAGCCCACTTTCTCTTCTC |
| HAUT27 | NA | AACTGCTGAAATCTCCATCTTA | TTTTATGTTCATTTTTTGACTGG |
| ILSTS029 | L37252 | TGTTTTGATGGAACACAGCC | TGGATTTAGACCAGGGTTGG |
| ILSTS30 | L37212 | CTTAGACAACAGGGGTTTGG | CTGCAGTTCTGCATATGTGG |
| INRA175 | NA | TGATGAGGATGGATGCTAAACT | CTGCAAATAAGAAAACTGAATAAA |
| JMP29 | U30893 | GTATACACGTGGACACCGCTTTGTAC | GAAGTGGCAAGATTCAGAGGGGAAG |
| Maf209 | M80358 | GATCACAAAAAGTTGGATACAACCGTGG | TCATGCACTTAAGTATGTAGGATGCTG |
| MAF36 | M80519 | CATATACCTGGGAGGAATGCATTACG | TTGCAAAAGTTGGACACAATTGAGC |
| McM152 | L39825 | CCTAGAAGCCTGGCTAAAATGTG | GGAACTCTCATAGTTTCCCACTCC |
| MILSTS76 | 9982 | TGGCAGGCAGGTTCTTTAGC | TTCAGATTCACTCAGACAGC |
| OarkP6 | AF223411 | GCCCTGTGTCTCGTGTAACTCAC | CCACAGGGTTGCAAAGAATCA |
| SR-CRSP07 | NA | TCTCAGCACCTTAATTGCTCT | GGTCAACACTCCAATGGTGAG |
| SR-CRSP24 | NA | AGCAAGAAGTGTCCACTGACAG | TCTAGGTCCATCTGTGTTATTGC |
| SR-CRSP25 | NA | AACTATAACGGGAAGGAGTCTGG | AGGTTGTAGGAGTCGGACACAG |
| TGLA10 | NA | CTAAATTTATCCCACTGTGGCTCTT | CAATCTGCAGTAGCATACATCCTTG |
| TGLA122 | NA | CCCTCCTCCAGGTAAATCAGC | AATCACATGGCAAATAAGTACATAC |
| URB058 | U21788 | GTAAGGCTCTTTGAGGGTTAGG | GCTTAGAAGTTTCTGTGCTGTC |

Table S2. Details of primer sequences, product size and genbank accession numbers.

| Gene | Primer name | Primer sequence (5’-3’) | Primer position | Product size (pb) | GENBANK accession numbers |
| --- | --- | --- | --- | --- | --- |
| *Tlr1* | TLR1a-F  TLR1a-R  TLR1b-F  TLR1b-R | ATGCCTGACATCCTCTCACTATCTCTTCTAAGCCAGGTTGAGTTCTTGC  ATAACAGAATAAGGAGCATCCC TCTGTGTAACGTACTTCTGCTGC | exon 2  exon 2  exon 2  exon 2 | 1243  1012 | MK802330-> MK802331 |
| *Tlr2* | TLR2aCapra-F  TLR2aCapra-R  TLR2b-F  TLR2b-R  TLR2c-F  TLR2c-R | AATCAGCGCGTTCACGGAAG GTGACTTTTCTAACTTTGCCTG  GGAGACGTTGACAATACGGA CTTGCCAGGAACGAAGTCTC  TTTGCTCCTGTGACTTCCTG GCCACTCCAGGTAGGTCYTG | exon 2  exon 2  exon 2  exon 2  exon 2  exon 2 | 987  1123  687 | MK802323-> MK802325 |
| *Tlr4* | TLR4a-F  TLR4aCapra-R  TLR4b-F  TLR4b-R  TLR4c-F  TLR4c-R | CCACCTCTCCACCTTGATAC GAACGGAGCTCCAGTGCAGG  GTGCAACCTGACCATTGAAC TCCTGGCTCGAGTAGATGAC  GACCTCTCTAAGTGTCAACTGG CTGCTGTTCCTTCTGGACTC | exon 3  exon 3  exon 3  exon 3  exon 3  exon 3 | 750  1215  982 | MK802326-> MK802329 |
|  |  |  |  |  |  |

Table S3. Summary of 7 SNPs genotyped using the KASPar SNP genotyping system.

| Gene | SNP^1^ | AA site^2^ | Codon Change | Syn/Nonsyn^3^ |
| --- | --- | --- | --- | --- |
| *Tlr1* | TLR1 1742 A/G | 597 | AAC(Asn) > AGC(Ser) | Non Syn |
| *Tlr2* | TLR2 103 C/G | 51 | ACA (Thr) > ACG (Thr) | Syn |
|  | TLR2 878 A/G | 310 | CAG (Gln) > GAG (Glu) | Non Syn |
|  | TLR2 1727 A/G | 593 | ACC (Thr) > GCC (Ala) | Non Syn |
| *Tlr4* | TLR4 564 A/G | 300 | AAT (Asn) > GAT (Asp) | Non Syn |
|  | TLR4 1799 A/G | 711 | AGG (Arg) > AGA (Arg) | Syn |
|  | TLR4 1824 A/G | 720 | GCC (Ala) > ACC (Thr) | Non Syn |

^1^ SNP position relative to start of the Alpine ibex sequence produced with primers in Table S2. ^2^ Position of SNP codon relative to aligned goat amino acid sequence. ^3^ Denotes whether the SNP is synonymous or non-synonymous.

**Table S4.** **Association between *Brucella melitensis* infection prevalence (presence or absence) in Alpine ibex (N= 237) from the Bargy massif (French Alps) and *Slc11A1* genotype**, age (linear and quadratic terms), sex, capture year and socio-spatial units. Parameter estimates are provided with their unconditional standard errors (SE) and 95% confidence intervals (CI), after model averaging (models with ΔAICc < 7; Table S6 and S7). Estimates where 95% CI do not overlap zero are highlighted in bold. Females from SSU2 sampled in 2012 were used as the reference category.

| Parameter | Estimate | Unconditional SE | IC 2.5% | IC 97.5% |
| --- | --- | --- | --- | --- |
| (Intercept) | -2.71 | 1.31 | -5.27 | -0.15 |
| slc11A1 : A324/A330 | -0.24 | 0.32 | -0.86 | 0.39 |
| slc11A1 : A330/A330 | **-2.36** | **1.14** | **-4.60** | **-0.13** |
| Age | 0.41 | 0.28 | -0.13 | 0.95 |
| Age^2 | -0.02 | 0.02 | -0.06 | 0.01 |
| 2017 | 0.06 | 0.64 | -1.19 | 1.30 |
| 2016 | -0.07 | 0.59 | -1.23 | 1.10 |
| 2015 | 0.25 | 0.65 | -1.03 | 1.52 |
| 2014 | -1.69 | 0.87 | -3.39 | 0.02 |
| 2013 | -1.63 | 0.82 | -3.24 | -0.03 |
| SSU4 | **2.99** | **0.77** | **1.49** | **4.49** |
| SSU3 | **1.88** | **0.60** | **0.69** | **3.07** |
| SSU1 | -0.14 | 0.73 | -1.57 | 1.29 |
| SSU5 | 1.50 | 0.64 | 0.23 | 2.76 |
| Sex (Male) | **-0.90** | **0.32** | **-1.53** | **-0.27** |

**Table S5. Association between *Brucella melitensis* infection prevalence (presence or absence) in Alpine ibex (N=146) from the Bargy massif (French Alps) and neutral multi-locus heterozygosity (MLH) or *Tlr* genotype**, age (linear and quadratic terms), sex, capture year and socio-spatial units. Parameter estimates are provided with their unconditional standard errors (SE) and 95% confidence intervals (CI)), after model averaging (models with ΔAICc<7; Table S6 and S7). Estimates where 95% CI do not overlap zero are highlighted in bold. Females from SSU2 sampled in 2012 were used as the reference category.

| Parameter | Estimate | Unconditional SE | IC 2.5% | IC 97.5% |
| --- | --- | --- | --- | --- |
| (Intercept) | -2.28 | 1.46 | -5.14 | 0.57 |
| MLH | 0.61 | 0.84 | -1.04 | 2.26 |
| nb of Tlr1a : 1 copy | **-1.90** | **0.82** | **-3.52** | **-0.29** |
| Heterozygous TLR1 | **1.90** | **0.82** | **0.29** | **3.52** |
| nb of Tlr2a : 1 copy | -0.34 | 0.89 | -2.08 | 1.39 |
| nb of Tlr2a : 2 copies | -0.30 | 0.87 | -2.00 | 1.40 |
| nb of Tlr2b : 1 copy | -0.21 | 0.54 | -1.26 | 0.84 |
| nb of Tlr2b : 2 copies | 1.52 | 1.49 | -1.40 | 4.44 |
| nb of Tlr2c : 1 copy | -0.20 | 0.48 | -1.14 | 0.74 |
| nb of Tlr2c : 2 copies | 1.52 | 1.76 | -1.93 | 4.96 |
| Heterozygous TLR2 | -0.19 | 0.42 | -1.02 | 0.63 |
| nb of Tlr4a : 1 copy | -0.80 | 0.52 | -1.82 | 0.22 |
| nb of Tlr4a : 2 copies | -1.23 | 0.91 | -3.02 | 0.55 |
| nb of Tlr4b : 1 copy | -0.79 | 0.56 | -1.89 | 0.31 |
| nb of Tlr4b : 2 copies | -0.31 | 0.89 | -2.07 | 1.44 |
| nb of Tlr4c : 1 copy | 0.40 | 0.48 | -0.54 | 1.35 |
| nb of Tlr4c : 2 copies | 1.37 | 0.86 | -0.32 | 3.06 |
| Heterozygous TLR4 | -0.56 | 0.59 | -1.71 | 0.59 |
| SSU5 | 1.04 | 0.81 | -0.55 | 2.62 |
| SSU1 | -0.68 | 1.40 | -3.43 | 2.07 |
| SSU3 | **1.96** | **0.80** | **0.40** | **3.53** |
| SSU4 | **2.61** | **0.88** | **0.89** | **4.33** |
| 2013 | 0.31 | 0.72 | -1.11 | 1.72 |
| 2014 | 0.35 | 0.67 | -0.97 | 1.66 |
| Age | 0.28 | 0.28 | -0.28 | 0.84 |
| Age^2 | -0.01 | 0.02 | -0.05 | 0.03 |
| Sex (Male) | -0.95 | 0.41 | -1.75 | -0.15 |
